# Physical Performance, Cardiovascular Health and Psychosocial Wellbeing in Older Adults Compared to Oldest-Old Residential Seniors

**DOI:** 10.3390/ijerph19031451

**Published:** 2022-01-27

**Authors:** Alice Minghetti, Lars Donath, Henner Hanssen, Ralf Roth, Eric Lichtenstein, Lukas Zahner, Oliver Faude

**Affiliations:** 1 Department of Sport, Exercise and Health, University of Basel, 4052 Basel, Switzerland; henner.hanssen@unibas.ch (H.H.); ralf.roth@unibas.ch (R.R.); e.lichtenstein@unibas.ch (E.L.); lukas.zahner@unibas.ch (L.Z.); oliver.faude@unibas.ch (O.F.); 2 Department of Intervention Research in Exercise Training, German Sport University Cologne, 50933 Cologne, Germany; l.donath@dshs-koeln.de

**Keywords:** aging, older adults, nursing homes, quality of life, strength

## Abstract

Background: This study analyzed physical, cardiovascular, and psychosocial health in different age groups at the far end of the lifespan. Methods: Sixty-two residential seniors participated in this cross-sectional study and were assigned according to age to either the older adults (*n* = 27; age: 74.8 (3.6); f: 23) or the oldest-old group (*n* = 35; age: 87.2 (5.0); f: 28). Gait speed, functional mobility, handgrip strength, and pulse wave velocity (PWV) were measured. Additionally, questionnaires to assess quality of life were applied. Mean between-group differences (Δ) and Hedge’s *g* with 95 % confidence intervals were calculated. Results: Oldest-old had moderately lower handgrip strength (Δ = −31.3 N, 95% CI [−66.30; −1.65], Hedge’s *g* = 0.49 [−0.97; 0.03]) and relevant lower gait speed than the older adults (Δ = −0.11 m/s [−0.28; 0.05], *g* = 0.34 [−0.89; 0.20]). All other physical parameters showed trivial differences. Very large effects were found in PWV in favor of the older adults (Δ = −2.65 m/s [−3.26; −2.04], *g* = −2.14 [−2.81; −1.36]). The questionnaires showed trivial to small differences. Conclusion: We found small differences in physical as well as psychosocial health between age groups with large inter-individual variance. Large differences were found in arterial stiffness, which increases with age. Exercise programs in nursing homes should consider physical, psychosocial, and cardiovascular variables more than age.

## 1. Introduction

As life expectancy of our population increases, the importance of healthy aging and increasing the number of disability-free years becomes all the more relevant. People aged 80 years and older are projected to triple in number within 30 years, representing the most rapidly growing age group [1]. Consequently, the preservation of health and physical and psychosocial function in the oldest-old will become a crucial challenge for the healthcare system and general public. Switzerland spends more than 10.4 billion Swiss francs on nursing homes or home-based care per year, tending for more than 150,000 seniors in these settings [2]. To design adequate and tailored interventions to the specific and individual needs of residential seniors, potential differences concerning various bodily systems between the age groups at the far end of the lifespan have to be examined and understood.

On a neuromuscular level, the biological aging process negatively affects motor function and sensory systems [3,4]. Motor units undergo a process of reduction and adaptation due to progressive motoneuron death and fiber denervation [5]. This degenerative process negatively influences the capacity to produce force adequately. From the sixth decade onward, the loss of lean muscle mass and, more pronounced, muscular strength is substantial: in healthy adults in their seventh and eighth decade of life, this can result in a 20–40% loss of isometric strength compared to young adults in their thirties and forties. In the ninth decade, it can lead up to a 50% loss [3,6]. Such substantial declines in physical performance not only negatively impact functional ability and, in turn, daily life and independent living, but also lead to a high risk of falling [7]. The risk of sustaining a fall increases with age: while in Switzerland seniors between 65 and 79 years of age are at a 25% risk of falling in the course of a year, the number rises to 33% in octogenarians [8]. Furthermore, falls in the elderly are directly linked to declines in functionality, institutionalization, and mortality [9]. 

Apart from neuromuscular deteriorations, prominent changes due to aging and inactivity occur in the structural and mechanical properties of the vascular system, resulting in increased arterial stiffening [10]. The stiffening and dilatation of large arteries is a degeneration of the arterial wall. This process is detectable by an increased pulse wave velocity (PWV) and the development of systolic hypertension [11]. About 50% of people aged 60 years and older are affected by hypertension and, in general, cardiovascular disease remains the leading cause of death worldwide [12]. 

These biological aging processes have a negative impact also on psychosocial wellbeing and therefore quality of life. Impairments and limitations in one’s functional mobility are not only correlated to mortality and institutionalization [13], but also to the reduction of social activities and interactions and a decreased quality of life [7]. Quality of life, therefore, is strongly dependent on physical functioning. Studies were able to show lower health-related quality of life in nursing home residents than in seniors living at home, which is mainly caused by lower physical activity levels in nursing homes [14]. Physical autonomy is of utmost importance in old age if one wishes to live in a healthy and fulfilling mental state. 

From a public health perspective, understanding the health status and functionality at the far end of a lifespan might be necessary in order to tailor specific health promotion programs to support high quality of life in old age, especially in residential care. Most available information on physical and psychosocial health comes from seniors between 60 and 80 years of age, omitting older individuals. Few studies have provided insight into the oldest-old (≥81 years old) [5], and reference values for physical performance parameters in these late stages of life are therefore largely lacking. These age groups nevertheless are of special interest since the few existing studies have shown that the decline in functionality accelerates in the ninth decade of life [5,13,15]. Our study therefore examined functional mobility, cardiovascular health, and psychosocial wellbeing in residential seniors, comparing the older adults (≤80 years old) with the oldest-old (≥81 years old). We additionally examined whether there were any associations between outcome parameters in the different health domains and compared our data to available reference values. 

## 2. Methods

### 2.1. Study Design and Study Population

We asked all nursing homes and senior living facilities in the city of Basel-Stadt to participate, and included all homes which were interested. In total, five senior residences in the canton Basel-Stadt, Switzerland, including 62 seniors (age: 81.2 (7.6) years; f: 51) participated in this cross-sectional study. Seniors living in the participating facilities of at least 65 years of age were included. Excluded were seniors suffering from one or more of the following illnesses: chronic and/or congenital heart failure, peripheral neuropathy, peripheral arterial occlusive diseases, diabetes mellitus or a diagnosis of cognitive illness such as Alzheimer’s disease or dementia. Participants were recruited and screened by medical staff in the respective nursing homes. For the present analysis, we divided the enrolled participants into two age groups: either the older adults (≤80 years old) or the oldest-old (≥81 years old) group [5]. The study was approved by the local ethics committee (Ethikkommission Nordwest- und Zentralschweiz, ethical approval number: 2018-01123) and all participants signed a written informed consent after having been informed about the study. 

### 2.2. Procedures

We conducted all measurements in the respective nursing homes. A team of experienced assessors adhering to standard operation procedures conducted the tests. Nursing home staff was included in the test standardization for the questionnaires. We performed all measurements in the morning and the patients were asked to refrain from physical exercise, eating, as well as drinking caffeine or alcohol 12 h prior to testing, as these factors can influence cardiovascular measurements. We collected anthropometric data (weight and height) prior to the physical and cardiovascular tests to calculate body mass index (BMI).

### 2.3. Short Physical Performance Battery

We assessed functional mobility using the three subtests from the Short Physical Performance Battery (SPPB), a reliable and validated protocol to objectively measure lower extremity physical performance and identify older individuals at risk of poor lower-body function. The SPPB is highly predictive for disability, hospitalization, institutionalization, and mortality in community-dwelling elders, with lower scores indicating higher levels of disability [16,17]. The sub-tests consist of usual walking speed over 4 m, the five repetition chair rising test, and three individual balance tests. Participants did not use walking aids such as canes, walking frames, or another person for support in any of the tests. 

For gait speed, we recorded the average velocity over 10 m with timing gates (Witty System) in a single- as well as dual-task situation. We used the 4 m split time for the SPPB score analysis. Participants started 1 m before the gate and walked in their habitual speed (i.e., “as if you were walking down the street to walk to a store”) to 1 m after the last timing gate. During dual-task, seniors counted backwards out loud starting from 50 in the first and from 70 in the second walk over the same distance. Participants performed each test twice and we used the faster time for analysis. If one person was not able to walk the 4 m distance, this person received 0 points. Scores between 1 and 4 were assigned to the time needed to walk 4 m (1 point: >8.7 s.; 2 points: 6.21–8.7 s.; 3 points: 4.82–6.20 s.; 4 points: ≤4.81 s.) [16]. 

Participants performed the repeated chair rising test on a force plate (Leonardo Mechanography^®^ GRFP LT, Pforzheim, Germany) with a 46 cm high, locked bench. Participants were asked to fully stand up and sit back down as fast as possible for a total of five repetitions. Participants were thereby not allowed to push off the bench with their hands. The force plate records time per repetition as well maximal power during every stand-to-sit cycle. Total time for the completion of all five stand-to-sit cycles as well as the relative peak power (W/kg) were calculated using raw data exports. Inability to complete all five repetitions, or requiring more than 60 s resulted in 0 points. Points between 1 and 4 were attributed accordingly: 1 point: >16.7 s.; 2 points: 13.7–16.69 s.; 3 points: 11.2–13.69 s.; 4 points: ≤11.19 s [16].

For the balance assessment, three different stances had to be performed for a total of 10 s: (a) side-by-side stance, with feet placed in a hip-wide distance; (b) semi-tandem stance where one foot of choice was placed with the side of the heel touching the big toe of the other foot, and; (c) tandem stance, where the heel of one foot was placed in front of and touching the toes of the other foot. If participants were not able to hold the stance for 10 s, or if they did not attempt the stance, they received 0 points. Holding the side-by-side and semi-tandem stance for the entire duration was scored with 1 point each. Holding the tandem stance for 10 s was attributed with 2 points, holding the stance for 3 to 9.99 s with 1 point, and less than 3 s or not at all with 0 points [16]. 

### 2.4. Handgrip Strength

We assessed handgrip strength using the Leonardo Mechanography GF^®^ (Novotec Medical GmbH, Pforzheim, Germany). Cut-off points predictive for survival analysis are available [18]. Participants were asked to hold their dominant hand in a 90-degree angle and to squeeze the sensor as hard as possible for 5 s. Participants performed two valid measurements with 1 min rest in between. Measurements were counted as invalid if the participants used both hands, lost their grip, or stopped squeezing the device during the 5 s. We extracted peak strength in N from raw data and used the mean of two valid measurements for statistical analysis. 

### 2.5. Cardiovascular Health 

The measurement of arterial stiffness parameters using the oscillometric method has been validated [19] and is in good agreement with the conventional tonometric method [20]. Markers of central hemodynamics (central systolic and diastolic blood pressure), augmentation index corrected for 75 bpm (AIx@75) and carotid–femoral pulse wave velocity (PWV) were obtained using a Mobil-O-Graph^®^ PWA Monitor device (I.E.M. GmbH, Stoberg, Germany) with integrated ARCSolver^®^ software. We placed the blood pressure cuff on the left upper arm while the patient was in a seated position after a 10 min resting period. Participants undertook all measurements in a fasting state. We asked them to refrain from physical exercise, eating, as well as drinking caffeine or alcohol 12 h prior to measurement. Twenty-three patients (37%) took antihypertensive medication. These patients refrained from taking this medication the day prior to and of the measurement. We used the mean of three valid measurements for data analysis. 

### 2.6. Questionnaires

Participants filled out three questionnaires with the help of the local nursing staff. Nursing staff read the questions and the possible answers, and asked participants to choose the most appropriate one. As all questionnaires provide examples and reflect situations of daily life, the study participants had no difficulty understanding the questions or giving accurate responses. 

The General Health Questionnaire (SF-36) is a validated and reliable questionnaire consisting of eight categories yielding a summary on physical as well as mental health [21]. The physical health includes four scales (General Health, Physical Functioning, Physical Limitations, Bodily Pain) and a total of 21 items. The mental health dimension is composed of four scales (Emotional Limitations, Vitality, Mental Health, Social Functioning) and 14 items. A higher score in all scales represents higher general health. 

The Assessment of Quality of Life (AQoL-8D) is a reliable and valid questionnaire to assess quality of life [22], especially in seniors due to its high sensitivity to particular dimensions of physical and psycho-social health. The questionnaire examines the following dimensions in a total of 35 items, all of which are independently representative for the individual dimension of health: independent living, pain, mental health, life satisfaction, coping, relationships, self-worth, and senses. We did not integrate the “senses” dimension in the applied questionnaire as assessment of sensory system and disease status was not an aim of our study. The lower the sum in all dimensions, the better is the psycho-social state of an individual.

The Falls Efficacy Scale (FES) consists of 16 questions and is a widely accepted and applied tool to assess individual concern about falling in the elderly, with excellent reliability and validity [23]. The higher the score (range: 16–64), the more severe is the subjective concern of falling. 

### 2.7. Statistical Analysis

We present the data for both groups as means with standard deviations (SD). We calculated independent *t*-Tests for mean between-group differences (Δ) and Hedge’s *g* with 95% confidence intervals. We estimated confidence intervals by bootstrapping with 5000 re-samples. We interpret the P values as a continuous measure of compatibility of the data with the statistical model and not relative to an arbitrary significance threshold [24]. Hedge’s *g* is a variation of Cohen’s *d* that corrects for biases due to small sample sizes. Effect sizes were interpreted as trivial (*g* < 0.2) small (0.2 ≤ *g* < 0.5), moderate (0.5 ≤ *g* < 0.8) and large (*g* ≥ 0.8) effects [25]. We performed these analyses by means of estimationstats.com [26]. Additionally, we calculated Pearson’s correlation analysis with 95% confidence intervals to measure the association between two variables whereby the correlation coefficient was interpreted as small (0.10 ≤ *r* ≥ 0.29), moderate (0.30≤ *r* ≥0.49), large (0.50 ≤ *r* ≥ 0.69), very large (0.70 ≤ *r* ≥ 0.89), and extremely large (*r* ≥ 0.90) [27]. We calculated the correlations by means of the jamoviStats software. 

## 3. Results

### 3.1. Study Population

Twenty-seven individuals belonged to the older adult group (age: 74.8 (3.6) years; BMI: 27.2 (3.6) kg/m^2^; female: 23) and 35 to the oldest-old group (age: 87.2 (5.0) years; BMI: 26.6 (4.7) kg/m^2^; female: 28).

### 3.2. Physical Functioning

**Figure A1:**
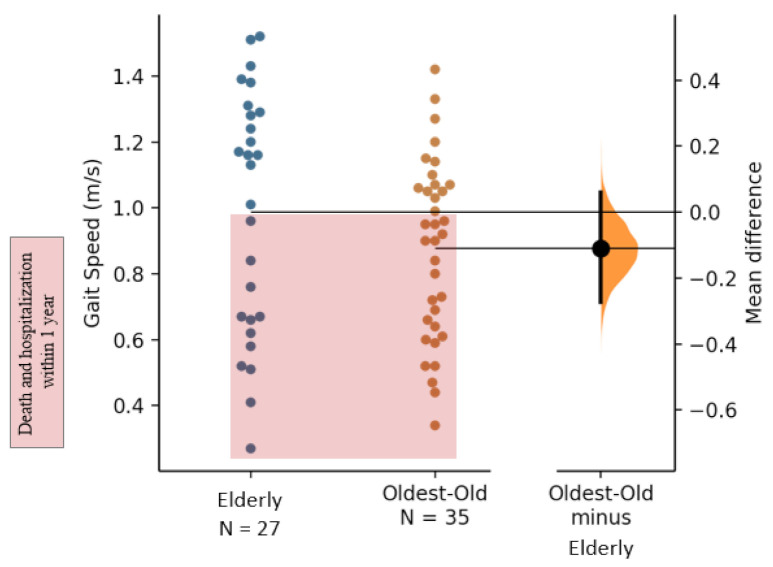
Individual datapoints of the older adults compared to oldest-old in single-task gait speed (m/s). Individuals in the red zone are at risk of death and hospitalization within one year according to Abellan van Kan et al. (2009).

Handgrip strength was higher in the older adults compared to the oldest-old (Δ = 31.3 N [1.7; 66.3], *g* = 0.49 [−0.03; 0.97]) with both age groups being on average slightly above risk threshold (216 N) [18]. We observed a small, but on average clinically relevant difference in single- as well as dual-task gait speeds in favor of the older adults (0.34 *< g* < 0.37). However, trivial as well as large effects were also compatible with the data (Figure A1). Effect sizes for between-group differences in all other physical functioning parameters ranged from trivial to small (0.11 *< g* < 0.37) with wide confidence intervals (Table 1).

### 3.3. Cardiovascular Health

**Figure A2:**
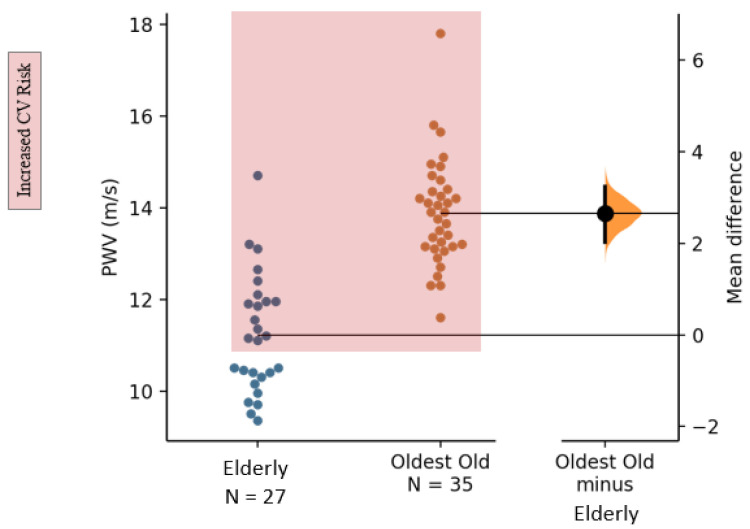
Individual datapoints of the older adults compared to oldest-old in PWV (m/s). Individuals in the red zone are at increased cardiovascular risk (Reference Values for Arterial Stiffness, 2010).

The oldest-old showed higher PWV (Δ = 2.65 m/s [2.04; 3.26], *g* = −2.14 [1.36; 2.81;]) as well as central systolic blood pressure values (Δ = 6.9 mmHg [−2.9; 15.4], *g* = 0.36 [−0.17; 0.88]) than the oldest-old. The 95% CI show that small as well as large effects were also compatible with the data (Figure A2). Central diastolic blood pressure (*g* = 0.06) as well as augmentation index (*g* = 0.32) showed trivial to small differences with wide confidence intervals (Table 1). 

### 3.4. Psychosocial Wellbeing

We found trivial differences between age groups in the total scores of the three questionnaires (0.12 < *g* < 0.19) with wide confidence intervals (Table 1). The self-worth dimension of the AQoL-8D was the only dimension which showed moderate effects in favor of the oldest-old (Δ = −1.12 [−2.05; −0.30], *g* = −0.63 [−1.12; −0.15]). All other dimensions showed only trivial differences in perception of physical and mental health between age groups (see Table A1 for all dimensions). 

Data are presented as mean with standard deviation (SD). Between-group differences are depicted (mean differences and hedge’s g) with 95% confidence intervals [95% CI]. SPPB = short physical performance battery; CRT= repeated chair rising test; cSBP = central systolic blood pressure; cDBP = central diastolic blood pressure; AIx@75 = augmentation index corrected for 75 beats per minute; PWV= pulse wave velocity; SF-36 = General Health questionnaire; AQoL-8D = Assessment of Quality of Life questionnaire; FES = Falls Efficacy Scale.

### 3.5. Correlations

**Figure A3:**
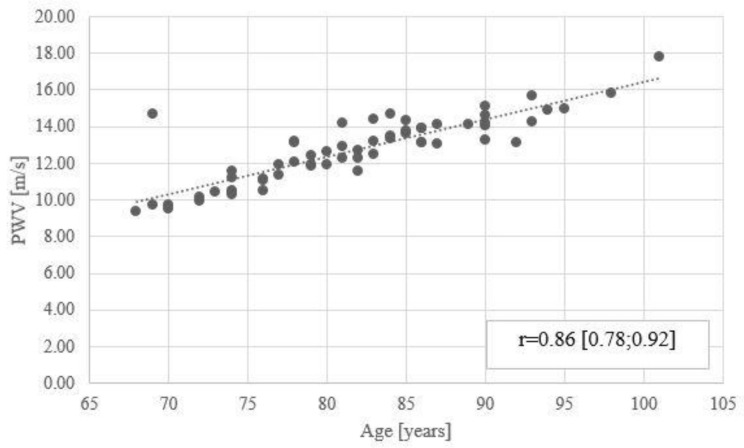
Correlation between age (years) and PWV (m/s).

**Figure A4:**
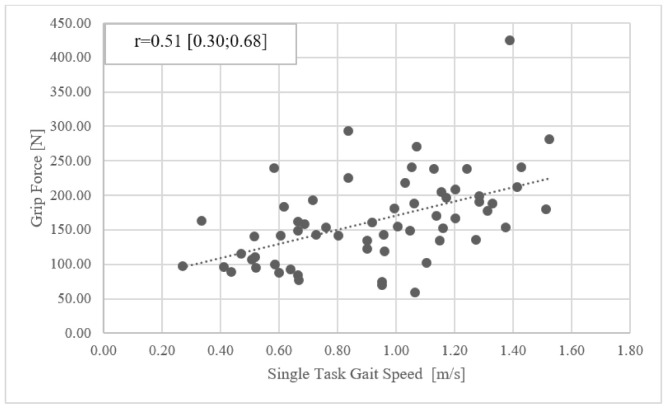
Correlation between handgrip strength (N) and single-task gait speed (m/s).

**Figure A5:**
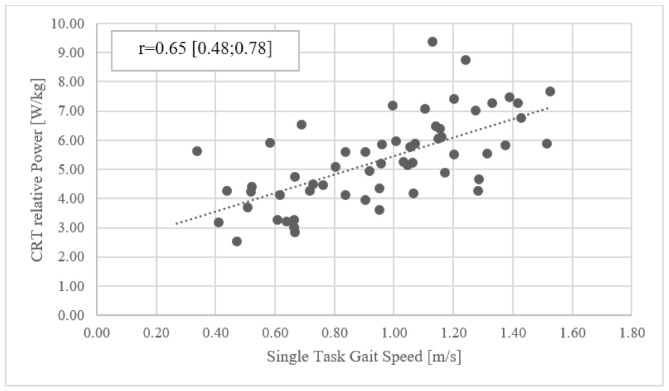
Correlation between relative power in CRT (W/kg) and single-task gait speed (m/s).

We observed a very large positive correlation between age and PWV (*r* = 0.86 [0.78; 0.92]) (Figure A3). All other correlations between physical functioning, cardiovascular health or psychosocial wellbeing and age showed only trivial to small effects with wide confidence intervals. Moderate to large correlations were found between grip strength and gait speed in the single- (*r* = 0.51 [0.30; 0.68]) as well as in dual-task situation (*r* = 0.55 [0.35; 0.71]) (Figure A4). We also observed a large correlation between gait speed and relative power in the lower extremities in the chair rising test (single-task: *r* = 0.65 [0.48; 0.78]; dual-task: *r* = 0.69 [0.52; 0.80]) (Figure A5). We found trivial correlations between cardiovascular health and physical functioning parameters as well as moderate correlations between psychosocial parameters and single-task (AQoL: *r* = −0.38 [−0.58; −0.15]; FES: *r* = −0.41 [−0.60; −0.18]) and dual-task gait speed (AQoL: *r* = −0.44 [−0.62; −0.20]; FES: *r* = −0.41 [−0.60; −0.17]). The correlation matrices are depicted in Table 2. 

## 4. Discussion

The present study examined physical, psychosocial, and cardiovascular health indices of older adults and the oldest-old of our society. Our results revealed that the age groups show relevant group differences in some physical functioning parameters, with the most-pronounced age-related differences manifesting on the cardiovascular level. Psychosocial wellbeing was similar in both age groups. 

Physical decline is not linear, but accelerates with age [28]. In our population, age did not correlate strongly with most physical performance variables as we observed higher performance of the older adults only in few parameters. SPPB scores were similar in both age groups and higher than established norm values for primary health care patients 70 years and older in all subtests, except the repeated chair rising test [29]. The older adults were stronger solely in handgrip strength, which is correlated to bone mineral density and is applied to assess frailty and disability [30,31]. Our data therefore suggests that the strength in the oldest-old is compromised, which potentially indicates impaired bone health and a resulting higher frailty. Mean values in the older adults were slightly over the cut-off point for mobility limitations in community-dwelling older people (174 N for females, 258 N for males) while the oldest-old fell below that cut-off [32]. In total, 71% of the participants were below their respective threshold, indicating higher mobility limitations and an elevated risk of adverse events in the measured population [18,32]. Interestingly, even though the older adult group showed better mean values, handgrip strength did not correlate with age, implying that age in our population of seniors is not the primary cause of the differences in strength, and that other factors such as use of grip in daily life and strength training should be considered. 

Older adults showed superior performance in gait speed compared to the oldest-old (Figure A1). This difference may have clinical implications as this parameter is oftentimes applied to assess adverse outcomes in primary preventive care measures, and cut-off points according to habitual walking speed can be used to assess the risk [33]. According to the literature, gait speed below 1.0 m/s may lead to death and hospitalization within 1 year [33]. Walking speed showed only a small to moderate correlation with age in our examined population, adding further notion to the interplay of a variety of confounders, which may impact physical functioning beyond age alone. Daily activity levels as well physical health at admission are relevant factors which need to be considered in order to further understand the aging process and how living in nursing homes affects the human body. We are not able to make conclusive assumptions as to if and to what extent the degenerative process occurred since institutionalization, as physical performance at committal was not established. Nonetheless, our findings show that age alone is not the primary cause of physical decline in our population, implying that functional mobility and, in turn, independent living cannot be attributed solely to aging. 

Therapists in Swiss nursing homes perform activities in a group setting, and oftentimes do not differentiate or create a selection mechanism due to a lack of resources [2]. Physical functionality and strength should, therefore, be assessed individually in order to tailor effective exercise programs. By applying a simple and fast testing battery, such as the SPPB and handgrip strength, the relevant parameters can be assessed efficiently and accurately. These values would allow a tailored and specific approach according to residents’ physical needs regardless of age in the form of selected gait, balance, and strength training according to the performance level. 

While differences in physical performance were minimal between age groups, central systolic blood pressure as well as PWV were elevated in the oldest-old. In line with the scientific literature [34], this indicates higher cardiovascular risk with increasing age in our population. Cardiovascular aging is an important health determinant with arterial stiffness being an independent predictor of cardiovascular outcomes such as myocardial infarction, cognitive decline, stroke, as well as hormonal and metabolic dysfunctions [34,35]. Higher fitness is associated with reduced arterial stiffness in sedentary as well as in active populations [36]. In adult populations, research shows beneficial effects of physical activity on vascular health in dependence of exercise intensity [37]. Nonetheless, the effects of exercise on arterial stiffness in elderly patients are conflicting due to a lack of research in this field. Certain studies show that mild to moderate aerobic exercise reduces arterial stiffness in 50-year old individuals [38], while other authors found no reduction in PWV in 60-year olds at risk of cardiovascular disease after a high-intensity exercise intervention [39]. This implies reduced vascular adaptability with increasing age. This might be due to functional and structural properties of the vasculature. Regular exercise, applied long-term, can improve both vascular properties and provoke changes in structure and function [39]. Accordingly, life-long activity and its continuation even in old age become all the more relevant. Nonetheless, long-term effects of regular exercise in older adults and oldest-old residentials need to be examined in order to assess the effect sizes and to define reference values, which are limited to an upper range of 70 years of age [40]. In relation to normative data (10.9 m/s for septuagenarians), our data shows a continued increase of PWV with progressive age (11.2 and 13.9 m/s), implying an elevated risk for cardiovascular mortality. Starting at about 50 years of age, PWV has been shown to increase exponentially with age as previously summarized in a consensus document on arterial stiffness [40]. In fact, all participants in the oldest-old group show increased cardiovascular risk, independently of physical functioning (Figure A2). This finding undermines how age has a direct effect on vascular function. How the cardiovascular system adapts to exercise in octogenarians and nonagenarians should be a topic of further research in order to find applicable, safe, and effective training modalities in nursing homes. 

Psychosocial wellbeing was similar between age groups. We found moderate correlations between psychosocial variables and lower extremity function in residential seniors, indicating that better physical functioning leads to lower fear of falling as well as better quality of life. Fear of falling was slightly higher in the oldest-old, but not classified as high risk [23]. Both age groups showed high values in all SF-36 dimensions, which speaks in favor of the care they receive in their daily life. Our population shows higher values independently of age in psychosocial wellbeing and quality of life than norm values established in other populations of individuals 85 years and older [41]. Observations recording social interactions of nursing home residents reveal that only 10% of their time awake (approximately 100 min.) is spent by interacting with other people, which includes nursing staff and caretakers [42]. Interestingly, the oldest-old showed higher values in self-worth than the older adults. This could in part be attributed to higher individual affective and motivational personality systems such as dispositional optimism [43]. Whereas the older adults are possibly reminiscing their lost abilities, the oldest-old show contentment and acceptance and therefore do not perceive their physical health as a limiting factor “anymore”, which might beneficially affect their self-worth and mental health. Exercise programs in community settings have shown how improvements of quality of life after regular exercise go in line with physical improvements, and therefore possess high potential to impact residents in residential care [44,45].

Considering the biological aging processes, its occurrence and degree notably differs between individuals, and is strongly dependent on lifestyle, with physical activity levels playing an important role. Exercise interventions in the oldest-old have shown promising results, as the body is still capable of adapting to exercise stimuli even in very old and sedentary populations [46]. Average muscle fiber size, independent of physical ability, is preserved in old age [5], thus allowing specific exercise programs to target the oldest and inactive members of society and benefitting their health. According to observations in nursing homes, residentials spend solely 1% of the total time awake in active motion (taking steps), which corresponds to less than 10 min per day [42]. The systemic stimulus is therefore very low in everyday life. Exercise interventions targeting nursing home patients have shown improvements in disability status as well as combating late-life depression [47], but compared to non-residential seniors, they remain considerably less active. This sedentary lifestyle may weaken self-efficacy, adding to the already passive behavior, which feeds the vicious cycle of inactivity in residential seniors [48].

Exercise programs, which promote social activities and interaction between residents and encourage performing habitual tasks, should be included in daily activities [44,45]. Strength and functional mobility parameters of residents should be used to create training groups, thus allowing the content to address their needs more specifically. According to the ability of the different groups, the sessions should target endurance in daily movement patterns such as standing, sitting, and walking in combination with balance tasks and strengthening exercises to different degrees of intensity and/or training volume. This could strengthen functional movements crucial for independent living in a group setting without under- or overexerting residents on an individual level. To make further statements about the aging process in older adults and oldest-old, and the role as well as importance of exercise, longitudinal studies are necessary [49,50,51]. Our study shows that, in our examined population of residential seniors, inter-individual differences in a variety of physical, vascular, and psychosocial indices become more predictive for successful aging. Considering the impact of the aging population on public health factors, our data provide comparative data on age groups which are rarely examined. Our data provide information on health aspects which should be considered when designing adequate intervention strategies for nursing home residents while considering their physical and psychosocial predispositions.

### 4.1. Methodological Considerations

There are some limitations of the study which we need to mention. To understand the aging process and its impact on strength, cardiovascular health, and quality of life, longitudinal analyses are necessary. The sample size of the study is small due to the strict exclusion criteria. We made the age cut-off point according to Naro, Venturelli, Monaco, Toniolo, Muti, Milanese, Zhao, Richardson, Schena, and Reggiani [5], while other literature sets different age cut-off points (i.e., oldest-old >100) [52]. To classify the eldest population group more precisely and especially for applying the information in the field, further studies are necessary. Our data do not allow general conclusions of the respective age groups as, on one hand, the age span within the groups is large, and on the other hand, we found large inter-individual differences. Nonetheless, we report important information and exploratory data that contribute to the knowledge in the field. As most of our subjects were female residents, one must be careful in generalizing the information to males. The socio-economic background, especially their past physical activity and lifestyle were not collected, which would have provided deeper understanding in their health status. Nevertheless, the study assessed relevant health parameters in a variety of domains in very old residential seniors, which were free of common illnesses accompanied by old age. We were able to provide data on the interplay of physical, cardiovascular, and psychosocial health variables in a vulnerable population. Reference values in such old populations are largely lacking; nevertheless, we were able to compare relevant parameters to established norms for adult populations. Further studies should measure nursing home residents at committal and on a regular basis thereafter to provide insight into how living in an environment which releases them from physical tasks affects functionality and health. 

### 4.2. Conclusions and Practical Applications

We found selective but small differences between older adults and oldest-old seniors in physical performance and psychosocial well-being and a large decline in vascular health with age, with notable inter-individual differences. We cannot attribute the findings solely to aging. Inter-individual differences in a variety of physical, vascular, and psychosocial indices were evident, and these parameters should be assessed regularly in order to tailor group-based exercise programs for nursing homes. The implementation and continuation of regular, group-based exercise programs with content based on physical performance parameters should therefore be a main goal in senior residences and nursing homes. Gait, strength, and balance exercises should be incorporated to different degrees in order to maintain or re-gain some independence and autonomy in old age. Bouts of aerobic exercise, in combination with gait training, should be encouraged to improve cardiovascular functioning, particularly in the oldest-old. Social aspects of exercise should be considered and interactions encouraged to maintain and improve psychosocial wellbeing and quality of life. Furthermore, reference values for physical as well as psychosocial health in older adults and oldest-old living in care settings should be a topic of further investigation.

Considering the aging population as well as inactive lifestyles in old age, our study provides important information for exercise interventions in older adults and oldest-old in order to properly design and tailor exercises and activity strategies which consider physical and psychosocial predispositions of the population. 

## Figures and Tables

**Table 1:** Results for the older adults and oldest-old groups in all parameters.

	Older Adults	Oldest-Old	*t*-Test			
	Mean (SD)	Mean (SD)	Mean Difference [95% CI]		Hedge’s g [95% CI]	
SPPB Gait Score	3.3 (1.0)	3.4 (0.9)	0.10	[−0.35; 0.59]	0.11	[−0.40; 0.63]
SPPB CRT Score	1.7 (1.1)	1.5 (0.9)	−0.25	[−0.76; 0.23]	−0.25	[−0.75; 0.26]
SPPB Balance Score	2.9 (1.3)	2.7 (1.1)	−0.24	[−0.82; 0.40]	−0.20	[−0.73; 0.32]
SPPB Total Score	7.9 (2.6)	7.5 (2.1)	−0.38	[−1.52; 0.86]	−0.16	[−0.68; 0.39]
Gait Speed Single-task (m/s)	0.99 (0.37)	0.88 (0.27)	−0.11	[−0.28; 0.05]	−0.34	[−0.89; 0.20]
Gait Speed Dual-task (m/s)	0.89 (0.39)	0.77 (0.31)	−0.12	[−0.28; 0.05]	−0.37	[−0.92; 0.17]
CRT (s)	18.4 (10.1)	19.0 (7.9)	0.66	[−5.46; 3.72]	0.08	[−0.56; 0.65]
Rel. Pmax CRT (W/kg)	5.4 (1.9)	5.2 (2.0)	−0.20	[−1.00; 0.54]	−0.13	[−0.66; 0.39]
Grip Strength (N)	181 (73)	150 (54)	−31.3	[−66.3; −1.7]	−0.49	[−0.97; 0.03]
cSBP (mmHg)	120 (17)	126 (20)	6.9	[−2.9; 15.4]	0.36	[−0.17; 0.88]
cDBP (mmHg)	81 (8)	82 (11)	0.6	[−4.1; 5.5]	0.06	[−0.42; 0.56]
AIx@75 (%)	29.0 (11.1)	32.3 (9.6)	3.35	[−1.94; 8.37]	0.32	[−0.21; 0.85]
PWV (m/s)	11.2 (1.3)	13.9 (1.2)	2.65	[2.04; 3.26]	2.14	[1.36; 2.81]
SF-36: Total Score	76.5 (16.6)	73.2 (18.5)	−3.38	[−11.80; 5.35]	−0.19	[−0.67; 0.35]
AQoL-8D: Total Score	61.6 (12.8)	60.1 (13.4)	−1.54	[−8.0; 4.9]	−0.12	[−0.39; 0.61]
FES	22.9 (8.0)	24.3 (8.5)	1.38	[−2.87; 5.28]	−0.16	[−0.37; 0.63]

**Table 2:** Correlation matrix of all measured variables.

	AIx@75 [%]	PWV [m/s]	Grip [N]	Gait ST [m/s]	Gait DT [m/s]	CRT [s]	CRT [W/kg]	SPPB Balance	SPPB Gait	SPPB CRT	SPPB Total	SF-36	AQoL
PWV [m/s]	0.34 [0.13; 0.55]												
Grip [N]	−0.12 [−0.36; 0.13]	−0.22 [−0.45; 0.03]											
Gait ST [m/s]	−0.09 [−0.34; 0.16]	−0.21 [−0.43; 0.05]	0.51 [0.30; 0.68]										
Gait DT [m/s]	−0.14 [−0.38; 0.12]	−0.21 [−0.44; 0.05]	0.55 [0.35; 0.71]	0.95 [0.91; 0.97]									
CRT [s]	0.10 [−0.17; 0.35]	0.23 [−0.03; 0.46]	−0.24 [−0.47; 0.02]	−0.41 [−0.60; −0.16]	−0.43 [−0.62; −0.19]								
CRT [W/kg]	−0.19 [−0.43; 0.07]	−0.23 [−0.46; 0.03]	0.54 [0.33; 0.70]	0.65 [0.48; 0.78]	0.69 [0.52; 0.80]	−0.46 [−0.64; −0.23]							
SPPB Balance	0.06 [−0.20; 0.30]	−0.09 [−0.34; 0.16]	0.38 [0.14; 0.58]	0.41 [0.18; 0.60]	0.40 [0.16; 0.59]	−0.40 [−0.60; −0.16]	0.16 [−0.11; 0.40]						
SPPB Gait	−0.02 [−0.27; 0.23]	−0.01 [−0.26; 0.24]	0.35 [0.11; 0.55]	0.78 [0.66; 0.86]	0.69 [0.52; 0.80]	−0.24 [−0.47; 0.02]	0.57 [0.37; 0.72]	0.30 [0.06; 0.51]					
SPPB CRT	−0.16 [−0.40; 0.09]	−0.17 [−0.41; 0.08]	0.32 [0.07; 0.52]	0.49 [0.27; 0.66]	0.43 [0.20; 0.62]	−0.65 [−0.78; −0.47]	0.56 [0.36; 0.72]	0.25 [0.00; 0.47]	0.41 [0.17; 0.60]				
SPPB Total	−0.05 [−0.29; 0.21]	−0.13 [−0.37; 0.13]	0.47 [0.25; 0.65]	0.74 [0.60; 0.83]	0.67 [0.51; 0.79]	−0.60 [−0.74; −0.40]	0.55 [0.35; 0.71]	0.75 [0.62; 0.85]	0.74 [0.59; 0.83]	0.72 [0.58; 0.83]			
SF-36	0.01 [−0.24; 0.26]	−0.09 [−0.33; 0.16]	0.10 [−0.15; 0.34]	0.26 [0.01; 0.48]	0.30 [0.05; 0.51]	−0.29 [−0.51; −0.03]	0.28 [0.02; 0.50]	0.16 [−0.09; 0.40]	0.06 [−0.19; 0.31]	0.27 [0.02; 0.48]	0.22 [−0.03; 0.45]		
AQoL	−0.21 [−0.44; 0.04]	−0.07 [−0.32; 0.18]	−0.25 [−0.47; 0.00]	−0.38 [−0.58; −0.15]	−0.44 [−0.62; −0.20]	0.29 [0.03; 0.51]	−0.47 [−0.65; −0.24]	−0.01 [−0.26; 0.24]	−0.23 [−0.45; 0.04]	−0.30 [−0.51; −0.06]	−0.23 [−0.45; 0.03]	−0.64 [−0.76; −0.46]	
FES	−0.15 [−0.32; 0.11]	0.04 [−0.21; 0.29]	−0.29 [−0.51; −0.05]	−0.41 [−0.60; −0.18]	−0.41 [−0.60; −0.17]	0.29 [0.04; 0.51]	−0.39 [−0.59; −0.15]	−0.11 [−0.35; 0.15]	−0.36 [−0.56; −0.12]	−0.33 [−0.53; −0.08]	−0.34 [−0.54; −0.10]	−0.57 [−0.72; −0.37]	0.56 [0.36; 0.71]

Data are shown as correlation coefficient (*r*) with 95% confidence intervals.

## Data Availability

All data generated or analyzed during this study are included in this published article and Appendix A.

## Abbreviations

AIx@75 = augmentation index corrected for 75 beats per minute; AQoL-8D = Assessment of Quality of Life Questionnaire; cDBP = central diastolic blood pressure; CRT = repeated chair rising test; cSBP = central systolic blood pressure; FES = Falls Efficacy Scale; PWV = Pulse Wave Velocity; SD = Standard deviation; SF-36 = General Health Questionnaire; SPPB = Short Physical Performance Battery.

## Appendix A

**Table A1:** Results for all dimensions of the applied psychosocial questionnaires. Data are presented as mean with standard deviation (SD). Between-group differences are depicted (mean differences and hedge’s g) with 95% confidence intervals [95% CI].

	Older Adults	Oldest-Old	*t*-Test			
	Mean (SD)	Mean (SD)	Mean Difference [95% CI]		Hedge’s g [95% CI]	
SF-36: General Health	67 (19)	64 (17)	−2.8	[−11.8; 6.4]	−0.15	[−0.68; 0.36]
SF-36: Physical Functioning	68 (28)	61 (27)	−6.3	[−19.5; 7.5]	−0.23	[−0.73; 0.28]
SF-36: Physical Limitations	77 (33)	75 (38)	−1.9	[−19.1; 16.5]	−0.05	[−0.51; 0.49]
SF-36: Emotional Limitations	83 (33)	79 (38)	−3.7	[−20.6; 14.8]	−0.10	[−0.56; 0.44]
SF-36: Vitality	76 (16)	68 (22)	−8.6	[−17.6; 0.8]	−0.43	[−0.91; 0.06]
SF-36: Mental Health	78 (12)	75 (17)	−2.5	[−9.6; 4.6]	−0.16	[−0.66; 0.33]
SF-36: Social Functioning	89 (18)	88 (15)	−0.7	[−8.4; 8.2]	−0.04	[−0.54; 0.49]
SF-36: Bodily Pain	75 (27)	74 (22)	−0.7	[−12.2; 13.1]	−0.02	[−0.53; 0.51]
AQoL: Independent Living	7.6 (3.3)	8.6 (3.3)	0.9	[−0.76; 2.50]	0.28	[−0.26; 0.74]
AQoL: Pain	5.6 (2.7)	5.7 (2.3)	0.10	[−1.21; 1.26]	0.04	[−0.49; 0.55]
AQoL: Mental Health	16.0 (3.6)	15.3 (3.9)	−0.62	[−2.43; 1.23]	−0.16	[−0.65; 0.34]
AQoL: Life satisfaction	8.4 (2.0)	8.0 (2.2)	−0.42	[−1.48; 0.55]	−0.20	[−0.70; 0.29]
AQoL: Self-worth	6.0 (1.9)	4.9 (1.7)	−1.12	[−2.05; −0.30]	−0.63	[−1.12; −0.15]
AQoL: Coping	6.2 (1.8)	5.8 (2.0)	−0.35	[−1.22; 0.67]	−0.18	[−0.67; 0.35]
AQoL: Relationships	11.8 (2.4)	11.7 (2.9)	−0.16	[−1.48; 1.13]	−0.06	[−0.60; 0.43]

SF-36 = General Health questionnaire; AQoL-8D = Assessment of Quality of Life questionnaire; FES = Falls Efficacy Scale.
